# Use of biomarkers of metals to improve prediction performance of cardiovascular disease mortality

**DOI:** 10.1186/s12940-024-01137-4

**Published:** 2024-11-07

**Authors:** Samuel D. Fansler, Kelly M. Bakulski, Sung Kyun Park, Erika Walker, Xin Wang

**Affiliations:** 1 Department of Biostatistics, School of Public Health, University of Michigan, Ann Arbor, MI USA; 2 Department of Epidemiology, School of Public Health, University of Michigan, M5523 SPH II, 1415 Washington Heights, Ann Arbor, MI 48109-2029 USA; 3 Department of Environmental Health Sciences, School of Public Health, University of Michigan, Ann Arbor, MI USA

**Keywords:** Metal mixtures, Cardiovascular Disease, Mortality, Machine learning, Environmental exposures, NHANES

## Abstract

**Background:**

Whether including additional environmental risk factors improves cardiovascular disease (CVD) prediction is unclear. We attempted to improve CVD mortality prediction performance beyond traditional CVD risk factors by additionally using metals measured in the urine and blood and with statistical machine learning methods.

**Methods:**

Our sample included 7,085 U.S. adults aged 40 years or older from the National Health and Nutrition Examination Survey 2003–2004 through 2015–2016, linked with the National Death Index through December 31, 2019. Data were randomly split into a 50/50 training dataset used to construct CVD mortality prediction models (*n* = 3542) and testing dataset used as validation to assess prediction performance (*n* = 3543). Relative to the traditional risk factors (age, sex, race/ethnicity, smoking status, systolic blood pressure, total and high-density lipoprotein cholesterol, hypertension, and diabetes), we compared models with an additional 17 blood and urinary metal concentrations. To build the prediction models, we used Cox proportional hazards, elastic-net (ENET) penalized Cox, and random survival forest methods.

**Results:**

420 participants died from CVD with 8.8 mean years of follow-up. Blood lead, cadmium, and mercury were associated (*p* < 0.005) with CVD mortality. Including these blood metals in a Cox model, initially containing only traditional risk factors, raised the C-index from 0.845 to 0.847. Additionally, the Net Reclassification Index showed that 23% of participants received a more accurate risk prediction. Further inclusion of urinary metals improved risk reclassification but not risk discrimination.

**Conclusions:**

Incorporating blood metals slightly improved CVD mortality risk discrimination, while blood and urinary metals enhanced risk reclassification, highlighting their potential utility in improving cardiovascular risk assessments.

**Supplementary Information:**

The online version contains supplementary material available at 10.1186/s12940-024-01137-4.

## Background

Cardiovascular disease (CVD) is the top cause of mortality in the United States and globally [1, 2]. Accurate prediction of CVD by identifying its top risk factors is crucial to effectively prevent this deadly and prevalent disease. Common CVD risk prediction methods, including the Framingham Risk Score, Reynolds Risk Score, and Pooled Cohort Equations, use demographic factors (age, sex, race/ethnicity), clinical measures (blood pressure, diabetes status, total cholesterol, high density lipoprotein (HDL) cholesterol), and in specific instances, markers of inflammation (high sensitivity C-reactive protein) and family history measures [3–6]. These models predict CVD with varying accuracy [7]. These approaches, however, do not include environmental factors such as metals, which may increase prediction accuracy.

This study aimed to examine the predictive value of mixtures of metals measured in the blood and urine on CVD mortality. Exposure to toxic metals, such as lead, cadmium, and mercury, is known to have lasting adverse effects on the cardiovascular system, contributing to conditions such as hypertension, coronary heart disease, stroke, and increased mortality [8, 9]. Beyond these well-known risk factors, epidemiological evidence indicates that other metals may also pose cardiovascular risks. For instance, exposure to non-essential metals like arsenic and nickel has been associated with cardiovascular events, including atherosclerosis and elevated blood pressure [10, 11]. Moreover, higher concentrations of copper and cobalt, although essential, have been linked to oxidative damage and inflammation, thereby raising the risk of cardiovascular diseases [12, 13]. However, the predictive value of metals other than lead, cadmium, and mercury has never been examined in the context of CVD prediction. To build on prior work [14], we included multiple urinary metals in our prediction model, along with blood lead, mercury, and cadmium, which have already been shown to improve CVD mortality prediction, to assess whether adding these additional metals could further enhance predictive performance. We benchmarked our prediction findings with the Framingham Risk Score, which used age, sex, blood pressure, diabetes status, smoking status, HDL cholesterol, and total cholesterol as predictors for coronary heart disease [3]. In this study, we refer to these predictors as “traditional predictors” because they are the commonly used risk factors from which we are trying to improve prediction performance. As a secondary analysis, we implemented machine learning techniques such as elastic-net and survival random forest to attempt to further improve prediction performance of our metal mixtures model.

## Methods

### Study population

The study sample included seven continuous United States National Health and Nutrition Examination Survey (NHANES) cycles collected between 2003 and 2004 and 2015–2016. NHANES is an annual cross-sectional survey that combines survey data, laboratory measures, and physical examinations to provide comprehensive nutrition and health data from a representative sample of the noninstitutionalized United States population [15]. Among 61,087 participants who participated in NHANES between the 2003–2004 and 2015–2016 cycles and had linked mortality data available, we restricted our sample to 21,933 individuals who were 40 years of age or older, since the risk of cardiovascular events is very low among those under 40 years of age [16]. We further excluded participants with missing information on metals and traditional predictors, leaving a total of 7,085 participants for the analysis. Participants provided written informed consent at the time of participation. This secondary data analysis is approved by the University of Michigan Institutional Review Board (HUM00195078).

### Metal measurements

Venous blood samples and urinary samples were collected at mobile exam centers by trained NHANES laboratory technologists and shipped to the Division of Laboratory Sciences, National Center for Environmental Health for analysis. Whole blood was analyzed for lead, mercury, and cadmium using inductively coupled plasma-dynamic reaction cell-mass spectrometry (ICP-DRC-MS) [17]. Urinary concentrations of cesium, molybdenum, thallium, cobalt, barium, lead, cadmium, uranium, tungsten, and antimony were also measured using ICP-DRC-MS [17]. Urinary mercury concentration was determined by flow injection cold vapor atomic absorption analysis in 2003–2004, and by ICP-DRC-MS in all following cycles [17]. Total urinary arsenic was measured by ICP-DRC-MS, while urinary dimethylarsinic acid and arsenobetaine were measured using high performance liquid chromatography coupled with ICP-DRC-MS [17]. NHANES protocols for quality assurance and quality control (QA/QC) follow the 1988 Clinical Laboratory Improvement Act standards [18]. Detailed protocol manuals for each biomarker analysis across all cycle years are publicly available online through the NCHS [17]. For example, in 2015–2016 reported QA/QC procedures included screening equipment for contamination, documenting maintenance, regular laboratory inspections, and equipment calibration [19]. Biomarker concentrations below the corresponding limit of detection (LOD) were imputed by NHANES as LOD/√2. The LODs for each metal across all included NHANES cycles are reported in Table S1. For this analysis, 17 metals with detection rates over 50% were selected. Metals concentrations were natural-log-transformed due to their skewed distribution and standardized based on the mean and standard deviation of the training data. To account for hydration status, urinary creatinine was measured using the Roche/Hitachi Modular P Chemistry Analyzer. Missing values for urinary creatinine were imputed to the mean creatinine value of 114.01 mg/dL. We used these predicted creatinine levels in covariate-adjusted hydration standardization [20]. 

### Cardiovascular disease mortality measures

NHANES data were linked with public-use mortality data from the National Death Index [15]. Participants were followed up through December 31, 2019. CVD mortality was identified using the *International Classification of Diseases*, *Tenth Revision* (ICD-10) codes. The ICD-10 codes used were 100–109, 111, 113, 120–151, and 160–169.

### Covariate assessments

Demographic variables such as age, smoking status, race/ethnicity, and sex were collected using NHANES self-administered questionnaires [15]. Age was considered as a continuous variable. For confidentiality purposes, age was top coded at 85 years old. Smoking status was categorized as 0 (not a current smoker) or 1 (current smoker). Race/ethnicity was categorized as White, Black, Hispanic (defined as Mexican American or other Hispanic), or Other Race. Sex was categorized as male or female.

Systolic blood pressure (SBP) and diastolic blood pressure (DBP) (in mmHg) were obtained in three separate measurements, with a fourth measurement taken in case of interrupted or incomplete measurements. We calculated mean SBP for our study by disregarding the first measurement and averaging the remaining measurements for each participant. Total cholesterol was measured enzymatically in serum. HDL cholesterol was measured via a magnesium/dextran sulfate solution. Body mass index (BMI) was measured by trained health technicians using weight in kilograms divided by height squared in meters. Diabetes was defined as self-reported physician diagnosis of diabetes mellitus, use of self‐reported antidiabetic medication, or hemoglobin A1c of 6.5% or higher. Hypertension was defined based on self-reported diagnosis, SBP of 140 mmHg or higher, DBP of 90 mmHg or higher, or the use of antihypertensive medications.

### Statistical methods

To ensure robust evaluation of our predictive model, we randomly divided our dataset into training (*n* = 3,542) and testing (*n* = 3,543) sets in a 1:1 ratio. The distributions of covariates between the testing and training datasets were described using mean and standard deviation for continuous covariates and number and frequency for categorical covariates. A chi-square test was used to test for differences in proportion of CVD mortality rates between the training and testing datasets.

Survival time was counted from the NHANES examination until the date of death for participants who died due to CVD. For participants who died from other causes, survival time was right-censored at the date of death. Surviving participants were right-censored at the last follow-up date (December 31, 2019).

We used Cox proportional hazards regression models to analyze the association between predictors and survival time in the training data. Our primary analysis progressed through four models. In model 1, we included the established traditional predictors from previous CVD risk scores, including age, race/ethnicity, smoking status, SBP, total cholesterol, HDL cholesterol, BMI, hypertension status, and diabetes status. In model 2, we included linear terms of the available blood metals from NHANES. The purpose of model 2 was to compare our results to previous findings that blood metals can improved CVD mortality prediction performance [14]. In model 3, we further included linear terms of the available urinary metals from the NHANES data to attempt prediction performance improvement. In model 4, we additionally included quadratic terms and pairwise interactions to account for potential non-linear functional forms and multiplicative interactions. To validate the model assumptions, we used Schoenfeld residuals to assess the proportional hazards assumption, Martingale residuals to evaluate the linearity of the predictors, and calculated the variance inflation factor (VIF) to check for multicollinearity.

As a secondary analysis, we built the prediction models using two machine learning methods—regularized elastic-net (ENET) and survival random forest methods—in the training data. ENET is a regularization technique that combines ridge regression and Least Absolute Shrinkage and Selection Operator (LASSO) regression that adds penalty terms to the model that introduces bias to decrease variance and attempt to improve prediction performance [21–23]. It also performs variable selection, shrinking the coefficients of less impactful variables to zero. The alpha parameter for the ENET models represents the weight of the L1 and L2 penalties used in training the model. Specifically, alpha takes values between 0 and 1, where 0 corresponds to only using L2 norm (ridge regression) and 1 corresponds to only using L1 norm (LASSO). This parameter was selected as the optimal weight that gave the best prediction performances on the testing data. The optimal lambda (overall penalty) was selected via 10-fold cross validation. More specifically, the model was fitted 11 times: once to obtain the sequence of lambda values, and 10 more times to fit the model with each fold excluded from the dataset. The excluded fold for each fit was then used to evaluate error, and lastly these errors were averaged to get the training error. Then the lambda value with the minimal training error was selected. To adjust for traditional CVD risk factors within the models, we constrained these variables against shrinkage. The ENET analyses included a model with traditional predictors (model 5), a model combining traditional predictors with blood metal variables (model 6), a model that added urinary metals (model 7), and a model that incorporated squared and interaction terms for all metals (model 8). ENET was performed using the R package “glmnet” [22–24]. 

Random forest is an ensemble machine learning method that combines numerous decision trees to account for overfitting and non-linearity [25]. Survival random forest extends this method to time-to-event data [26, 27]. We tuned hyperparameters, including the number of nodes variables at each split, via out-of-bag validation. Node sizes between 1 and 100 were tried. Various number of variables were tried around the neighborhood of total number of variables divided by 2. Because the random forest model is designed to consider non-parametric effects, we only trained a baseline model with the traditional predictors (model 9), a model adding the blood metals (model 10), and a model further including urinary metal variables (model 11). The R package “randomForestSRC” was used to perform the survival random forest [28]. 

The prediction performance of all models was evaluated using the testing dataset. The risk discrimination was measured using Harrell’s C-index [29–31]. The C-index is a value between 0 and 1 that measures concordance between observed and predicted survival times [29–31]. Additionally, we applied the continuous Net Reclassification Index (NRI) to evaluate the risk reclassification [32, 33]. The NRI calculates the proportions of cases that are correctly assigned a higher predictive probability and noncases that are assigned a lower probability with the inclusion of additional predictors.

In sensitivity analyses, we compared our models to the original Framingham Heart Study [3]. To do this, we calculated the Framingham Risk Score based on score sheets for men and women [3], then fit Cox proportional hazards models with the total score as a predictor and validated the model’s prediction performance using C-index. We then attempted to improve the C-index from the Framingham Study’s score by adding the blood and urinary metals to the model. We attempted to further improve prediction performance by adding the squared and interaction terms to account for non-linearity. We also assessed our models’ performance when restricting follow-up time to various lengths. Specifically, we fit all models to follow-up times exceeding 3 years and 5 years, respectively. Additionally, we adjusted for more potential confounders, including the poverty-to-income ratio (PIR) and healthy eating index (HEI) [34], in the Cox proportional hazards models. Furthermore, we evaluated the model’s performance by excluding 39 participants (15 from the training set and 24 from the testing set) with missing urinary creatinine data. Finally, to address the potential imbalance between CVD mortality and non-CVD mortality in the dataset, we applied the synthetic minority oversampling technique (SMOTE) to create a more balanced training dataset [35]. SMOTE uses a k-nearest-neighbor algorithm to oversample the minority class by generating synthetic samples based on existing minority instances. Specifically, we applied SMOTE with 5 nearest neighbors and targeted a 50/50 distribution between CVD mortality cases and non-CVD mortality cases. After applying SMOTE, our training data included 6,496 observations, of which 3,165 (48.7%) were CVD mortality cases, comprising both original and synthetic cases. SMOTE was not applied to the testing data to ensure that model performance was validated on the original, unaltered dataset. The “smotefamily” R package was used to perform the SMOTE.

All analyses were conducted using R, version 4.2.0 (www.R-project.org).

## Results

### Study sample characteristics

Characteristics of the training and testing datasets are shown in Table 1. In the training dataset (*n* = 3,542), the mean (SD) age of participants was 60 (12) years. Throughout 16 years of follow-up, there 211 cases of cardiovascular disease mortality (mortality rate = 3.7 per 1000 person-year). Similar distributions of all characteristics were observed in testing dataset (*n* = 3,543).

**Table 1:** Characteristics of study participants in the National Health and Nutrition Examination Survey with complete data, randomly split into testing and training sets

Characteristic	Training data (*n* = 3,542)	Testing data (*n* = 3,543)
**Follow-up characteristics**		
CVD mortality, number of cases (%)	211 (6%)	209 (5.9%)
Follow-up time, mean (SD), years	8.9 (4.0)	8.7 (4.0)
**Baseline characteristics**		
Age, mean (SD), years	60 (12)	60 (13)
Race/ethnicity, number of participants (%)		
Non-Hispanic White	1,689 (48%)	1,667 (47%)
Non-Hispanic Black	713 (20%)	720 (20%)
Hispanic	850 (24%)	886 (25%)
Other race	290 (8.2%)	270 (7.6%)
Male, number of participants (%)	1,795 (51%)	1,748 (49%)
Current smoker, number of participants (%)	644 (18%)	659 (19%)
Systolic blood pressure, mean (SD), mmHg	129 (20)	129 (19)
Total serum cholesterol, mean (SD), mg/dL	199 (42)	200 (43)
HDL cholesterol, mean (SD), mg/dL	54 (17)	54 (17)
BMI categories, number of participants (%)		
Under- and normal weight (< 25 kg/m^2^)	921 (26%)	918 (26%)
Overweight (25–30 kg/m^2^)	1,294 (37%)	1,231 (35%)
Obese (≥ 30 kg/m^2^)	1,327 (37%)	1,394 (39%)
Hypertension, number of participants (%)	1,688 (48%)	1,721 (49%)
Diabetes, number of participants (%)	675 (19%)	641 (19%)
Blood lead, median (IQR), µg/dL	1.41 (0.93, 2.19)	1.41 (0.92, 2.23)
Blood mercury, median (IQR), µg/L	0.89 (0.46, 1.80)	0.88 (0.47, 1.72)
Blood cadmium, median (IQR), µg/L	0.35 (0.22, 0.62)	0.35 (0.21, 0.60)
Urinary total arsenic, median (IQR), µg/L	8.06 (3.89, 16.00)	7.64 (3.88, 17.03)
Urinary dimethylarsinic acid, median (IQR), µg/L	3.61 (2.00, 6.12)	3.56 (2.01, 6.29)
Urinary arsenobetaine, median (IQR), µg/L	1.30 (0.80, 6.36)	1.30 (0.82, 6.16)
Urinary cesium, median (IQR), µg/L	4.46 (2.74, 6.62)	4.53 (2.80, 6.86)
Urinary molybdenum, median (IQR), µg/L	40.20 (21.60, 68.66)	40.70 (21.60, 69.60)
Urinary thallium, median (IQR), µg/L	0.15 (0.09, 0.23)	0.15 (0.09, 0.24)
Urinary cobalt, median (IQR), µg/L	0.34 (0.21, 0.54)	0.35 (0.21, 0.56)
Urinary barium, median (IQR), µg/L	1.12 (0.56, 2.16)	1.17 (0.57, 2.24)
Urinary lead, median (IQR), µg/L	0.52 (0.28, 0.92)	0.54 (0.30, 0.94)
Urinary cadmium, median (IQR), µg/L	0.28 (0.14, 0.54)	0.29 (0.14, 0.57)
Urinary mercury, median (IQR), µg/L	0.34 (0.15, 0.76)	0.34 (0.15, 0.76)
Urinary uranium, median (IQR), µg/L	0.01 (0.00, 0.01)	0.01 (0.00, 0.01)
Urinary tungsten, median (IQR), µg/L	0.06 (0.03, 0.12)	0.07 (0.03, 0.13)
Urinary antimony, median (IQR), µg/L	0.05 (0.03, 0.09)	0.05 (0.03, 0.09)
Urinary creatinine, median (IQR), mg/dL	100.00 (58.00, 153.75)	105.00 (60.00, 155.00)

Note: CVD, cardiovascular disease; SD, standard deviation; HDL, high-density lipoprotein; BMI, body mass index; IQR, interquartile range

### Associations between metals and CVD mortality

Cox regression models for individual metals showed that only the metals measured in blood (lead, mercury, and cadmium) were significantly associated with CVD mortality (Table 2). The hazard ratios (HRs) for CVD mortality, corresponding to a one standard deviation increase in the log-transformed concentrations of metals, were as follows: blood lead (HR = 1.40, 95% confidence interval [CI]: 1.23, 1.59) and blood cadmium (HR = 1.21, 95% CI: 1.06, 1.38) were associated with increased hazard of CVD mortality. Conversely, blood mercury was associated with decreased hazard (HR = 0.80, 95% CI: 0.69, 0.92). There were no statistically significant associations between urinary metal concentrations and CVD mortality.

**Table 2:** Individual metal associations with cardiovascular disease mortality in the National Health and Nutrition Examination Survey

Metal^a^	Hazard Ratio^b^ (95% CI)	*P*
Blood lead	1.40 (1.23, 1.59)	< 0.001
Blood mercury	0.80 (0.69, 0.92)	0.001
Blood cadmium	1.21 (1.06, 1.38)	0.004
Urinary total arsenic	1.00 (0.99, 1.01)	0.512
Urinary dimethylarsinic acid	1.00 (0.99, 1.01)	0.957
Urinary arsenobetaine	1.00 (0.99, 1.01)	0.604
Urinary cesium	1.00 (0.99, 1.02)	0.384
Urinary molybdenum	1.00 (0.99, 1.01)	0.722
Urinary thallium	1.00 (0.99, 1.01)	0.604
Urinary cobalt	1.01 (1.00, 1.03)	0.137
Urinary barium	1.00 (0.99, 1.01)	0.768
Urinary lead	1.00 (0.99, 1.01)	0.824
Urinary cadmium	1.00 (0.99, 1.02)	0.563
Urinary mercury	1.00 (0.99, 1.01)	0.851
Urinary uranium	1.00 (0.99, 1.01)	0.889
Urinary tungsten	1.00 (0.99, 1.01)	0.831
Urinary antimony	1.00 (0.99, 1.01)	0.693

^**a**^ All metal concentrations were natural-log transformed and standardized, thus hazard ratios correspond to one standard deviation increase in the log scale of the metal concentrations

^**b**^ Hazard ratios were generated from separate Cox models for each chemical, adjusting for age, race/ethnicity, smoking status, systolic blood pressure, total cholesterol, high-density lipoprotein cholesterol, body mass index, hypertension status, and diabetes status. Urinary models were additionally adjusted for creatinine

### Predicting CVD mortality using primary cox models

Regarding the risk discrimination (Table 3), the Cox proportional hazards model using traditional continuous predictors alone (Model 1) yielded a C-index of 0.845. Incorporation of blood metals (Model 2) resulted in a minor increase in the C-index to 0.847. The addition of urinary metals (Model 3) decreased the C-index slightly to 0.843. Introducing quadratic and pairwise interactions for metals (Model 4) further reduced the C-index to 0.783. In terms of risk reclassification, the continuous NRI compared to Model 1 was 0.23 (95% CI: 0.09, 0.38) for Model 2, 0.21 (95% CI: 0.07, 0.35) for Model 3, and 0.35 (95% CI: 0.21, 0.48) for Model 4.

**Table 3:** Comparison of C-index and net reclassification index (NRI) across models. Models are predicting cardiovascular disease mortality in the testing dataset of the National Health and Nutrition Examination Survey

Model	C-index	Continuous NRI (95% CI)	# of Predictors^a^
**Cox Proportional Hazards Model**			
Model 1: Traditional Predictors^b^	0.845		10
Model 2: + Blood Metals^c^	0.847	0.23 (0.09, 0.38)	13
Model 3: + Urinary Metals^d^	0.843	0.21 (0.07, 0.35)	28
Model 4: + Metals quadratic/interaction terms^e^	0.773	0.35 (0.21, 0.48)	177
**Elastic-Net**			
Model 5: Traditional Predictors^b^	0.828		10
Model 6: + Blood Metals^c^	0.830	0.66 (0.52, 0.80)	12
Model 7: + Urinary Metals^d^	0.830	0.51 (0.37, 0.64)	14
Model 8: + Metals quadratic/interaction terms^e^	0.830	0.76 (0.62, 0.90)	13
**Survival Random Forest**			
Model 9: Traditional Predictors^b^	0.833		10
Model 10: + Blood Metals^c^	0.826	-0.18 (-0.32, -0.04)	13
Model 11: + Urinary Metals^d^	0.826	-0.49 (-0.63, -0.35)	28

^a^ Number of Predictors: refers to the total number of variables included in the model

^b^ Traditional predictors include age, race/ethnicity, smoking status, systolic blood pressure, total cholesterol, high density lipoprotein cholesterol, body mass index, hypertension status, and diabetes status

^c^ Blood metals include lead, mercury, and cadmium

^d^ Urinary metals include cesium, molybdenum, thallium, cobalt, barium, lead, cadmium, uranium, tungsten, antimony, mercury, arsenic, dimethylarsinic acid, and arsenobetaine

^e^ Metals quadratic/interaction terms indicate all possible quadratic and pairwise interactions between metals listed above

Schoenfeld’s global test indicated that the proportional hazards assumption was violated in Model 1 (*P* = 0.02), but not in Model 2 (*P* = 0.06), Model 3 (*P* = 0.27), and Model 4 (*P* = 0.18). Variance Inflation Factor (VIF) values were below 10 for all predictors in Models 1–3, except for urinary arsenic in Model 3 (VIF = 12.7), suggesting minimal multicollinearity. Martingale residuals showed minimal violation of the linearity assumption for continuous predictors (Figure S1).

### Predicting CVD mortality using secondary machine learning approaches

For the ENET model, the C-index showed slight improvement from the model with only traditional predictors (Model 5, C-index = 0.828) to the model including main effects for the metal concentrations (Model 6, C-index = 0.830) (Table 3). Blood lead, blood mercury, and urinary cesium were retained as nonzero predictors for CVD death (Table S2). The inclusion of quadratic and interaction terms for metals (Model 8) in the ENET model also produced a C-index of 0.830. Nonzero predictors retained in Model 8 included blood lead, blood mercury, and an interaction term for urinary thallium*urinary creatinine. Regarding risk reclassification, the continuous NRI for Model 6 compared to Model 5 was 0.66 (95% CI: 0.52, 0.80), for Model 7 compared to Model 5 it was 0.51 (95% CI: 0.37, 0.64), and for Model 8 compared to Model 5, it was 0.76 (95% CI: 0.62, 0.90).

The survival random forest model utilizing only traditional predictors (Model 9) achieved a C-index of 0.833 (Table 3). When blood metals were included alongside traditional predictors (Model 10), the model’s C-index decreased to 0.8326. The addition of urinary metals to traditional predictors also gave a C-index of 0.826 (Model 11). Non-positive continuous NRIs were observed for both Model 10 and Model 11.

### Sensitivity analyses

The model that incorporated the Framingham Risk Score as a predictor of CVD mortality yielded a C-index of 0.771 for men and 0.680 for women, showing a slight underperformance compared to the original study’s C-index of 0.730 for men and 0.760 for women, as noted in Table S3. The inclusion of main effects for blood metals in the model resulted in a modest improvement in the C-index to 0.776 for men and 0.707 for women. However, the addition of main effects for urinary metals led to a reduction in the C-index to 0.768 for men and 0.670 for women. Further incorporation of interaction and quadratic terms for metals decreased the C-index to 0.625 for men and 0.618 for women.

When restricting follow-up time to more than 3 and 5 years, the C-indices showed only a slight decrease across all models (Table S4). However, the continuous NRI significantly decreased for Models 2 and 4 when the follow-up time was restricted to more than 3 years.

Adjusting for PIR and HEI in the Cox proportional hazards models yielded similar results (Table S5).

When evaluating the model’s performance by excluding 39 participants with missing urinary creatinine data, the results remained consistent with those of the primary analysis (Table S6).

After applying the SMOTE to balance the training dataset, we observed similar improvements in predictive performance when incorporating metals as compared to the primary analysis (Table S7).

## Discussion

In this large, diverse sample of 7,085 U.S. adults aged 40 years and above with up to 16 years of follow-up, the incorporation of a combination of three metal concentrations in blood (lead, cadmium, and mercury) improved the predictive performance for CVD mortality compared to only using the traditional risk factors in terms of both risk discrimination and risk reclassification. However, additionally adding urinary metals to the model with traditional CVD risk factors and blood metals did not further improve the risk discrimination. Despite this, the inclusion of urinary metals enhanced risk reclassification metrics in both the Cox proportional hazards and ENET models.

Our findings are consistent with previous research with similar study samples. For example, Wang et al. utilized NHANES cycles from 1999 to 2012 and observed a C-index of 0.845 when predicting CVD mortality using the same traditional predictors as in our study [14]. The original Framingham Risk Score showed a sex stratified C-index of 0.74 for men and 0.77 for women [3]. When using the categorized risk scores from the Framingham study as a predictor for CVD mortality in the NHANES data, we obtained C-indices of 0.771 for men and 0.680 for women. Although our model with continuous predictors study did not stratify by sex, our C-index using the traditional predictors (including race/ethnicity and continuous age, SBP, HDL-C and total cholesterol) was noticeably larger (0.845). This is likely due to the larger and more diverse sample size that the NHANES dataset provides, as well as the precision gained from using continuous predictors. Specifically, the Framingham study consisted of a study sample of white participants aged 30 to 74 years old, while our study included a more representative sample of the U.S. population and only included adults aged 40 and older. In addition, we used their model to predict CVD mortality, while the model was originally designed to predict coronary heart disease risk [3]. 

There are few other studies investigating the efficacy of environmental factors on CVD mortality prediction. Previous research has found that particulate matter and Normalized Difference Vegetation Index improved C-index compared to only traditional variables when predicting stroke and myocardial infarction [36]. It has also been shown that adding lead, cadmium, and mercury measured in the blood to a model only including traditional predictors improved both C-index and net reclassification index for CVD mortality in NHANES data [14]. These findings indicate potential utility of environmental risk factors for CVD risk and mortality and emphasize the necessity of additional research in this area.

Although incorporating blood metals into the prediction model enhanced performance in a similar cohort, our findings indicate that the addition of urinary metals did not improve the predictive performance for CVD mortality in terms of risk discrimination. However, the inclusion of urinary metals did enhance risk reclassification in Cox proportional hazards and ENET models. There are several possible explanations for this discrepancy. Our analysis revealed no significant associations between urinary metals and CVD mortality, either individually or in a mixture model. The inclusion of predictors not strongly linked to the outcome is unlikely to enhance discrimination accuracy. Moreover, introducing fourteen urinary metals increased the model’s complexity. While this complexity may have led to overfitting, diminishing the discrimination accuracy, it appears to have improved the model’s ability to improve predicted risks in the right direction, thereby enhancing the NRI.

In our secondary analysis, we found that machine learning methods did not enhance CVD mortality prediction performance as measured by Harrell’s C-index compared to traditional methods. Among the prediction methods evaluated, the Cox proportional hazards model yielded the best results, both with only traditional predictors and with the inclusion of main effects from metals. ENET was developed to overcome limitations of ridge regression and LASSO, such as handling high correlations between predictors and addressing complex, non-linear relationships with the outcome [21]. Similarly, the random forest method is suited for complex prediction scenarios and for dealing with non-linear predictors [25]. However, because both methods are optimized for more complex modeling scenarios than those presented in this analysis, the Cox model appeared to be the most effective for this particular context. Additionally, the relatively low number of CVD mortality events in our dataset may have hindered the performance of the machine learning methods due to insufficient outcome events to train these models effectively.

Prediction of CVD mortality was the primary goal in this study, but we additionally evaluated the associations between individual metals and CVD mortality to better understand the relationship between our exposures and our outcome. Our findings confirm the associations of blood lead, blood cadmium, and blood mercury with mortality from previous studies [37–39]. Past research also shows an association between urinary cadmium and CVD mortality [40, 41], but was not observed in our analysis. Lead has been shown to have an association with lipoprotein disorders and atherosclerosis and blood cadmium has been linked to elevated blood pressure, another risk factor for CVD [42]. Associations between mercury and CVD are inconclusive, but dietary seafood intake could confound this association [42]. 

The findings of our current study extend the conclusions of our previous research, wherein we demonstrated that incorporating blood heavy metals into traditional CVD prediction models enhances predictive performance for CVD mortality [14]. Although adding urinary metals did not significantly enhance risk discrimination (C-index), it did improve risk reclassification (NRI). While these gains in predictive metrics are modest and may have limited real-world impact, even small improvements can have public health significance given the prevalence of cardiovascular disease. Clinically, these results suggest that integrating environmental exposure data into risk assessments could help better identify at-risk populations, particularly for populations with high environmental exposure, leading to more targeted preventive strategies and potentially more efficient allocation of healthcare resources. Our study had many limitations to consider. First, the ENET method produces biased coefficient estimates due to the penalty term it introduces for variable selection. Although we prefer unbiased estimates, the bias incurred from ENET was acceptable because we were only interested in prediction performance of our model as opposed to inference. Second, confounding continues to be a limitation in many observational studies, and ours is no exception. We attempted to control for confounding by adjusting for sociodemographic and lifestyle variables consistent with previous studies, but there are possibly more confounding variables that have not been accounted for that could affect the validity of past and current CVD prediction research. Third, NHANES uses complex survey weighting methods to ensure that each cycle is representative of the U.S. population [15]. Due to the challenges of incorporating survey weights into the machine learning models used in this study, we elected not to account for the NHANES survey design. As a result, our findings are generalizable to the study population within NHANES, but not necessarily to the broader U.S. population. Fourth, there is a lack of robust methods to statistically compare C-index values between different machine-learning models in survival analysis. Finally, our study attempted to improve CVD mortality prediction by adding a mixture of 17 metals to the model at once. It is difficult to determine which individual metals contributed the most to the Cox model with this method, because we did not examine the individual prediction efficacy of individual metals.

Our analysis also has several strengths. One such strength is the diversity of the study sample. The large sample size and a nearly nine-year average follow-up time gave us the power to build complex models to consider the predictive value of seventeen different metals on CVD mortality. The metal exposures are well quantified and were collected from both blood and urine to provide an accurate measurement of metal exposure from the study sample. Our study used a combination of classical statistical models and modern machine learning techniques to create and validate complicated metal mixtures prediction models. Our findings were robust to multiple train-test splits.

## Conclusions

In a study of a large sample, we observed that incorporating blood metal concentrations (lead, cadmium, and mercury) modestly improved the prediction performance for CVD mortality in both the Cox proportional hazards models and ENET models, compared to using traditional risk factors alone. While the addition of urinary metal concentrations did not enhance risk discrimination, it did improve risk reclassification metrics. Given the ongoing global burden of CVD mortality, our findings offer insights into potential predictors that could be considered to better identify and manage individuals at risk for CVD.

## Electronic supplementary material

Below is the link to the electronic supplementary material.

Supplementary Material 1

## Data Availability

No datasets were generated or analysed during the current study.

## Abbreviations

BMI: Body mass index
C-index: Concordance index
CVD: Cardiovascular disease
DBP: Diastolic blood pressure
ENET: Elastic-net
HEI: Heathy Eating Index
HDL: High-density lipoprotein
HR: Hazard ratio
ICD-10: International Classification of Diseases, Tenth Revision
ICP-MS: Inductively coupled plasma-mass spectrometry
LASSO: Least absolute shrinkage and selection operator
LOD: Limit of detection
NHANES: National Health and Nutrition Examination Survey
NRI: Net reclassification index
PIR: Poverty-to-income ratio
SBP: Systolic blood pressure
SMOTE: Synthetic minority oversampling technique
VIF: Variance inflation factor
