# Supplementary material for: Use of biomarkers of metals to improve prediction performance of cardiovascular disease mortality
Source: Environ Health. 2024 Nov 7;23:96. doi: 10.1186/s12940-024-01137-4 (PMC11542438; doi:10.1186/s12940-024-01137-4)
Supplement: Supplementary file 1 — Supplementary Material 1 [file 12940_2024_1137_MOESM1_ESM.docx]

**Use of Biomarkers of Metals to Improve Prediction Performance of Cardiovascular Disease Mortality**

**Supplementary Materials**

Samuel D. Fansler, Kelly M. Bakulski, Sung Kyun Park, Erika Walker, Xin Wang

**Table S1.** Lower limits of detection for included metal biomarker measurements for each cycle of the National Health and Nutrition Examination Survey, 2003-2016.

**Table S2.** Selected coefficients and beta estimates from elastic-net models. Models are predicting cardiovascular disease mortality in the training dataset of the National Health and Nutrition Examination Survey.

**Table S3**. C-index comparison across models using sex-stratified Framingham Risk Score. Models are predicting cardiovascular disease mortality in the testing dataset of the National Health and Nutrition Examination Survey.

**Table S4**. Comparison of C-index and Net Reclassification Index (NRI) across models using restricted follow-up times of 3 or more (3+) and 5 or more (5+) years.

**Table S5.** Comparison of C-index and Net Reclassification Index (NRI) in Cox proportional hazards models after additionally adjusting for poverty-to-income ratio and the healthy eating index. Models are predicting cardiovascular disease mortality in the testing dataset of the National Health and Nutrition Examination Survey.

**Table S6**: Comparison of C-index and Net Reclassification Index (NRI) in Cox proportional hazards models after excluding 39 participants with missing values in urinary creatinine.

**Table S7**: Comparison of C-index and Continuous Net Reclassification Index (NRI) across models using Synthetic Minority Oversampling Technique (SMOTE).

**Figure S1**. Martingale residuals of the Cox proportional hazards modeling with traditional and metal predictors.

| **Metal** | **NHANES Cycle** | | | | | | |
| --- | --- | --- | --- | --- | --- | --- | --- |
|  | **2003-2004** | **2005-2006** | **2007-2008** | **2009-2010** | **2011-2012** | **2013-2014** | **2015-2016** |
| Blood lead (ug/dL) | 0.283 | 0.255 | 0.255 | 0.255 | 0.250 | 0.070 | 0.070 |
| Blood mercury, total (ug/L) | 0.198 | 0.198 | 0.283 | 0.325 | 0.160 | 0.280 | 0.280 |
| Blood cadmium (ug/L) | 0.141 | 0.198 | 0.198 | 0.198 | 0.160 | 0.100 | 0.100 |
| Urinary cesium (ug/L) | NA | NA | NA | 0.066 | 0.120 | NA | 0.086 |
| Urinary molybdenum (ug/L) | 1.499 | NA | 0.919 | 0.920 | 0.990 | 0.800 | 0.800 |
| Urinary thallium (ug/L) | 0.016 | 0.016 | 0.016 | 0.015 | 0.020 | 0.018 | 0.018 |
| Urinary cobalt (ug/L) | 0.085 | 0.041 | 0.041 | 0.041 | 0.048 | 0.023 | 0.023 |
| Urinary barium (ug/L) | 0.212 | 0.113 | 0.113 | 0.120 | 0.100 | 0.060 | 0.060 |
| Urinary lead (ug/L) | 0.099 | 0.099 | 0.099 | 0.100 | 0.080 | 0.030 | 0.030 |
| Urinary cadmium (ug/L) | 0.042 | 0.042 | 0.042 | 0.042 | 0.056 | 0.036 | 0.036 |
| Urinary uranium (ug/L) | 0.001 | 0.002 | 0.002 | 0.002 | 0.003 | 0.002 | 0.002 |
| Urinary tungsten (ug/L) | 0.014 | 0.021 | 0.021 | 0.021 | 0.026 | 0.018 | 0.018 |
| Urinary antimony (ug/L) | 0.028 | 0.033 | 0.033 | 0.032 | 0.041 | 0.022 | 0.022 |
| Urinary mercury (ug/L) | 0.141 | 0.085 | 0.085 | 0.080 | 0.050 | 0.130 | 0.130 |
| Urinary arsenic, total (ug/L) | 0.600 | 0.740 | 0.740 | 0.740 | 1.250 | 0.260 | 0.260 |
| Urinary dimethylarsinic acid (ug/L) | 1.700 | 1.700 | 1.700 | 1.700 | 1.800 | 1.910 | 1.910 |
| Urinary arsenobetaine (ug/L) | 0.400 | 0.400 | 0.400 | 0.400 | 1.190 | 1.160 | 1.160 |

**Table S1.** Lower limits of detection for included metal biomarker measurements for each cycle of the National Health and Nutrition Examination Survey, 2003-2016.

All blood measures 2003-2010 and urinary measures excluding arsenic, dimethylarsinic acid, and arsenobetaine 2003-2008: calculated as LOD = (fill value of measures below LOD)/sqrt(2). Fill values sourced from NHANES documentation

All blood measures 2011-2016, urinary arsenic, dimethylarsinic acid, and arsenobetaine measures 2003-2016, and all other urinary measures 2009-2016: sourced from NHANES documentation

NA = cannot calculate from fill value because no measures in that cycle were below LOD (fill value and LOD not stated)

**Table S2.** Selected coefficients and beta estimates from elastic-net models. Models are predicting cardiovascular disease mortality in the training dataset of the National Health and Nutrition Examination Survey.

| **Metal Predictors** | **Beta Coefficient** | | |
| --- | --- | --- | --- |
|  | **Model 6^a^** | **Model 7^b^** | **Model 8^c^** |
| Blood Lead | 0.064 | 0.102 | 0.031 |
| Blood Mercury | -0.064 | -0.102 | -0.029 |
| Blood Cadmium | 0^d^ | 0 | 0 |
| Urinary Cesium | 0 | 0.001 | 0 |
| Urinary Creatinine | 0 | -0.051 | 0 |

^a^ Model 6 includes traditional predictors and blood metal concentrations.

^b^ Model 7 includes traditional predictors and urinary metal concentrations.

^c^ Model 8 includes traditional predictors and metals quadratic/interaction terms.

^d^ Coefficient estimates were shrunk to 0 by elastic-net.

**Table S3**. C-index comparison across models using sex-stratified Framingham Risk Score. Models are predicting cardiovascular disease mortality in the testing dataset of the National Health and Nutrition Examination Survey.

| **Models** | **C-index** | **# of Predictors** |
| --- | --- | --- |
| **Male (n=1,748)** | | |
| Model only using score^a^ | 0.771 | 1 |
| + Blood Metals^b^ | 0.776 | 4 |
| + Urinary Metals^c^ | 0.768 | 19 |
| + Metals quadratic/interaction terms^d^ | 0.625 | 169 |
| **Female (n=1,795)** | | |
| Model only using score^a^ | 0.680 | 1 |
| + Blood Metals^b^ | 0.707 | 4 |
| + Urinary Metals^c^ | 0.671 | 19 |
| + Metals quadratic/interaction terms^d^ | 0.618 | 169 |

^a^ Variables included in Framingham Risk Score include age, sex, blood pressure, diabetes status, smoking status, high-density lipoprotein cholesterol, and total cholesterol.

^b^ Blood metals include lead, mercury, and cadmium.

^c^ Urinary metals include cesium, molybdenum, thallium, cobalt, barium, lead, cadmium, uranium, tungsten, antimony, mercury, arsenic, dimethylarsinic acid, and arsenobetaine

^d^ Metals quadratic/interaction terms indicate all possible quadratic and pairwise interactions between metals listed above.

**Table S4.** Comparison of C-index and Net Reclassification Index (NRI) across models using restricted follow-up times of 3 or more (3+) and 5 or more (5+) years.

| **Model** | **C-index**  **(3+ Years)** | **C-index**  **(5+ Years)** | **Continuous NRI (95% CI)**  **(3+ Years)** | **Continuous NRI (95% CI)**  **(5+ Years)** |
| --- | --- | --- | --- | --- |
| **Cox Proportional Hazards Model** | | | | |
| Model 1: Traditional Predictors^a^ | 0.845 | 0.841 |  |  |
| Model 2: + Blood Metals^b^ | 0.855 | 0.843 | 0.02 (-0.03, 0.08) | 0.08 (-0.10, 0.25) |
| Model 3: + Urinary Metals^c^ | 0.850 | 0.832 | 0.22 (0.06, 0.38) | 0.04 (-0.13, 0.22) |
| Model 4: + Metals quadratic/interaction terms^d^ | 0.786 | 0.736 | 0.19 (0.04, 0.35) | 0.35 (0.16, 0.53) |
| **Elastic-Net** | | | | |
| Model 5: Traditional Predictors^a^ | 0.832 | 0.828 |  |  |
| Model 6: + Blood Metals^b^ | 0.835 | 0.829 | 0.67 (0.51, 0.82) | 0.53 (0.34, 0.72) |
| Model 7: + Urinary Metals^c^ | 0.834 | 0.829 | 0.68 (0.53, 0.84) | 0.55 (0.36, 0.74) |
| Model 8: + Metals quadratic/interaction terms^d^ | 0.833 | 0.829 | 0.74 (0.58, 0.89) | 0.53 (0.36, 0.71) |
| **Random Forest** | | | | |
| Model 9: Traditional Predictors^a^ | 0.833 | 0.812 |  |  |
| Model 10: + Blood Metals^b^ | 0.830 | 0.815 | 0.04 (-0.12, 0.19) | -0.44 (-0.63, -0.24) |
| Model 11: + Urinary Metals^c^ | 0.828 | 0.819 | -0.40 (-0.55, -0.24) | -0.52 (-0.70, -0.35) |

^a^ Traditional predictors include age, race/ethnicity, smoking status, systolic blood pressure, total cholesterol, high density lipoprotein cholesterol, body mass index, hypertension status, and diabetes status

^b^ Blood metals include lead, mercury, and cadmium.

^c^ Urinary metals include cesium, molybdenum, thallium, cobalt, barium, lead, cadmium, uranium, tungsten, antimony, mercury, arsenic, dimethylarsinic acid, and arsenobetaine.

^d^ Metals quadratic/interaction terms indicate all possible quadratic and pairwise interactions between metals listed above.

**Table S5.** Comparison of C-index and Net Reclassification Index (NRI) in Cox proportional hazards models after additionally adjusting for poverty-to-income ratio and the healthy eating index. Models are predicting cardiovascular disease mortality in the testing dataset of the National Health and Nutrition Examination Survey.

| **Model** | **C-index** | **Continuous NRI (95% CI)** | **# of Predictors** |
| --- | --- | --- | --- |
| **Cox Proportional Hazards Model** | | | |
| Model 1: Traditional Predictors^a^ | 0.872 |  | 12 |
| Model 2: + Blood Metals^b^ | 0.874 | 0.28 (0.12, 0.42) | 15 |
| Model 3: + Urinary Metals^c^ | 0.874 | 0.07 (-0.09, 0.23) | 30 |
| Model 4: + Metals quadratic/interaction terms^d^ | 0.756 | 0.35 (0.20, 0.49) | 179 |

^a^ Traditional predictors include age, race/ethnicity, smoking status, systolic blood pressure, total cholesterol, high density lipoprotein cholesterol, body mass index, hypertension status, and diabetes status. Poverty-to-income ratio and the healthy eating index are also adjusted.

^b^ Blood metals include lead, mercury, and cadmium.

^c^ Urinary metals include cesium, molybdenum, thallium, cobalt, barium, lead, cadmium, uranium, tungsten, antimony, mercury, arsenic, dimethylarsinic acid, and arsenobetaine.

^d^ Metals quadratic/interaction terms indicate all possible quadratic and pairwise interactions between metals listed above.

**Table S6**: Comparison of C-index and Net Reclassification Index (NRI) in Cox proportional hazards models after excluding 39 participants with missing values in urinary creatinine.

| **Model** | **C-index** | **Continuous NRI (95% CI)** | **# of Predictors** |
| --- | --- | --- | --- |
| **Cox Proportional Hazards Model** | | | |
| Model 1: Traditional Predictors^a^ | 0.845 |  | 10 |
| Model 2: + Blood Metals^b^ | 0.847 | 0.21 (0.08, 0.35) | 13 |
| Model 3: + Urinary Metals^c^ | 0.841 | 0.17 (0.03, 0.32) | 28 |
| Model 4: + Metals quadratic/interaction terms^d^ | 0.776 | 0.47 (0.32, 0.61) | 177 |

^a^ Traditional predictors include age, race/ethnicity, smoking status, systolic blood pressure, total cholesterol, high density lipoprotein cholesterol, body mass index, hypertension status, and diabetes status.

^b^ Blood metals include lead, mercury, and cadmium.

^c^ Urinary metals include cesium, molybdenum, thallium, cobalt, barium, lead, cadmium, uranium, tungsten, antimony, mercury, arsenic, dimethylarsinic acid, and arsenobetaine.

^d^ Metals quadratic/interaction terms indicate all possible quadratic and pairwise interactions between metals listed above.

**Table S7**: Comparison of C-index and Continuous Net Reclassification Index (NRI) across models using Synthetic Minority Oversampling Technique (SMOTE).

| **Model** | **C-index** | **Continuous NRI (95% CI)** | **# of Predictors** |
| --- | --- | --- | --- |
| **Cox Proportional Hazards Model** | | | |
| Model 1: Traditional Predictors^a^ | 0.845 |  | 10 |
| Model 2: + Blood Metals^b^ | 0.847 | 0.21 (0.08, 0.35) | 13 |
| Model 3: + Urinary Metals^c^ | 0.852 | 0.18 (0.03, 0.32) | 28 |
| Model 4: + Metals quadratic/interaction terms^d^ | 0.744 | 0.10 (-0.03, 0.25) | 177 |
| **Elastic-Net** | | | |
| Model 5: Traditional Predictors^a^ | 0.817 |  | 10 |
| Model 6: + Blood Metals^b^ | 0.821 | 0.49 (0.35, 0.63) | 12 |
| Model 7: + Urinary Metals^c^ | 0.819 | 0.42 (0.27, 0.56) | 14 |
| Model 8: + Metals quadratic/interaction terms^d^ | 0.740 | 0.15 (0.01, 0.29) | 174 |
| **Survival Random Forest** | | | |
| Model 9: Traditional Predictors^a^ | 0.811 |  | 10 |
| Model 10: + Blood Metals^b^ | 0.807 | 0.07 (-0.06, 0.19) | 13 |
| Model 11: + Urinary Metals^c^ | 0.806 | -0.18 (-0.29, -0.07) | 28 |

SMOTE was used to generate an oversampled training dataset with 3,165 cases of CVD deaths and 3,331 non-CVD deaths.

^a^ Traditional predictors include age, race/ethnicity, smoking status, systolic blood pressure, total cholesterol, high density lipoprotein cholesterol, body mass index, hypertension status, and diabetes status

^b^ Blood metals include lead, mercury, and cadmium.

^c^ Urinary metals include cesium, molybdenum, thallium, cobalt, barium, lead, cadmium, uranium, tungsten, antimony, mercury, arsenic, dimethylarsinic acid, and arsenobetaine.

^d^ Metals quadratic/interaction terms indicate all possible quadratic and pairwise interactions between metals listed above.


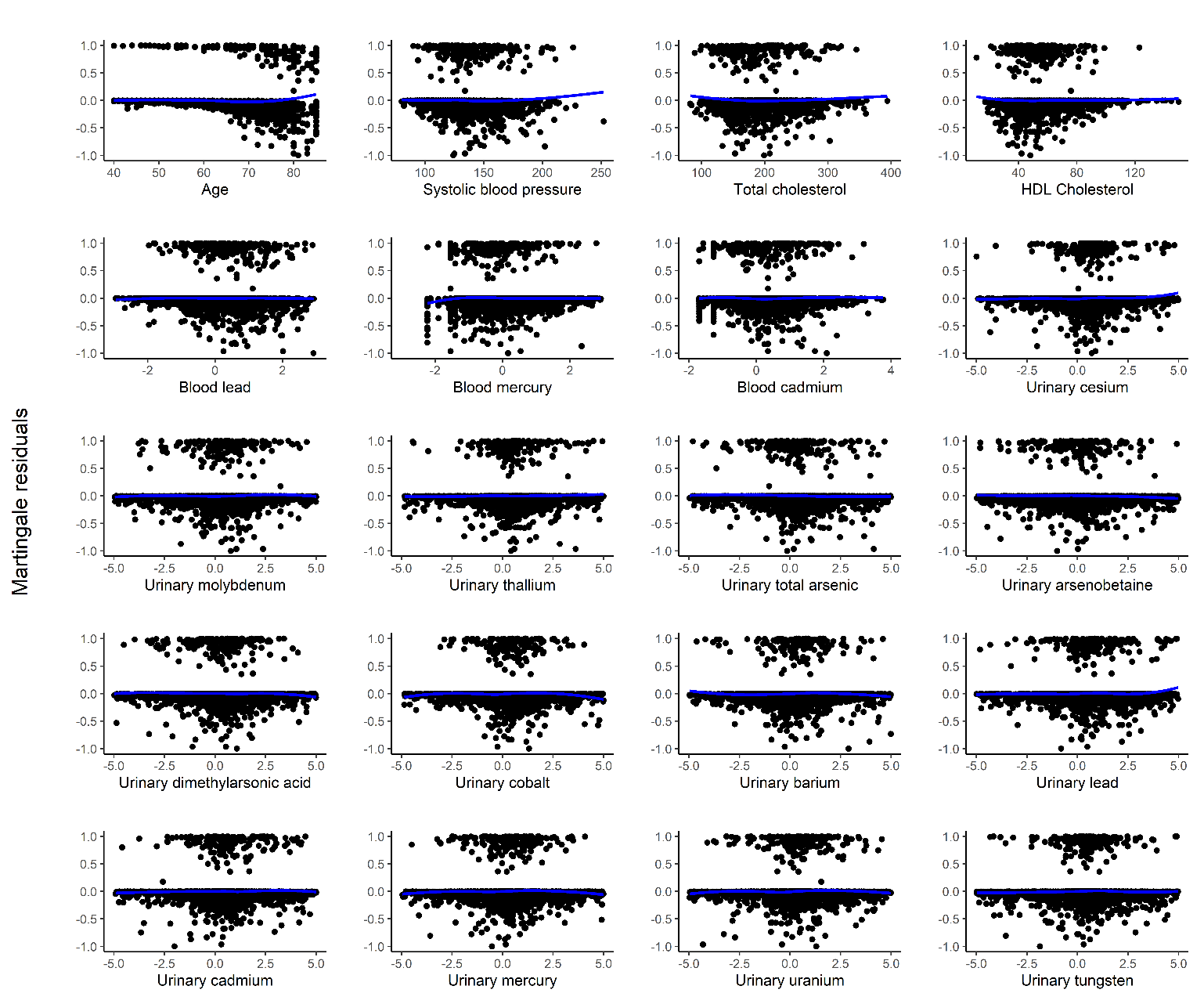


**Figure S1**. Martingale residuals of the Cox proportional hazards modeling with traditional and metal predictors.
